# A Royal Commission on Anæsthetics

**Published:** 1909-11-20

**Authors:** 


### The Hospital

A Journal of

The Medical Sciences and Hospital Administration.

New Series. No. 143, Vol. VI. [No. 1215, Vol. XLVII.] SATURDAY, NOVEMBER 20, 1909.

### A Royal Commission on Anæsthetics.

It is a recognised device in party politics to appoint a Royal Commission on any question, the decision or consideration of which it is desired indefinitely to postpone and suspend. The process, competently managed by the political wire-puller, is supposed to involve a delay of two, three, or more years, and to end in voluminous reports which nobody reads and impracticable proposals which nobody carries out. It is not with any such intent that a demand has of late years been voiced for a Royal Commission to consider many important questions tconnected with the administration of anæsthetics and with legislation thereon. Fortunately political cleavages in this country have not yet reached the point at which medicine or any other science is regarded as a protégé or an asset of any party; even anti-vivisectors are not quite all to be found in the ranks of one school of political thought. If such a Commission should eventually be appointed, it may be hoped that its findings will be arrived at without undue delay, though it is to be admitted that the number of points ripe for discussion is very large. If the signs of the times are not fallacious, the country is on the eve of a general election, and the present Government may be imagined to have little time to spare for non-partisan matters. When the election is over and things political are settling down, it may be suggested that this matter of anæsthetics should be pressed on the attention of the new Government, from whichever party its members are chosen.

The scope of the inquiry can hardly be indicated here. Several disputed or debateable points have been dealt with in these columns as they have arisen during the last year or two, and many of them are embodied in Dr. Hewitt's Anæsthetics Bill, which has aroused so much controversy and discussion both in the medical and dental professions. But within the last few days two inquests have been held in London upon the victims of anæsthetic misfortune, and at each certain points of great interest and importance have been raised. They may, therefore, fitly be alluded to as topical problems which should rightly come among the concerns of the hoped-for Commission. One of them can be dismissed very briefly, inasmuch as it is hardly an anæsthetic calamity at all, though to omit all mention of it would be a Responsibility of Silence such as we do not care to shoulder. As reported in the daily Press, it would appear that a boy died in St. Thomas's Hospital last week shortly after an operation upon glands in his neck, which lasted for four hours; the boy had undergone three previous operations for the same complaint. Now whether the anæsthetic or the operation had the greater share in causing (or accelerating) this death cannot be determined without a more complete knowledge of the case than is obtainable from a newspaper report. But, in any event, it may safely be said that in no circumstances should an operation of such duration be performed for this complaint. Whether the anæsthetist in such a case should decline to continue his co-operation, and when he should thus withdraw, are points very difficult to decide. But the surgeon whose action puts him in this predicament is one whose conduct is absolutely unjustifiable; his responsibility, at least, is clear and definite. It may be doubted whether such a case has ever been reported before, and it is to be hoped it may be very long before such another is heard of.

The other inquest was conducted by the City Coroner, Dr. Waldo, whose energy and ability in the investigation of anæsthetic fatalities have several times been the subject of comment in these columns. Fortunately, the reporters who exploit the public hunger for tragedy and sensation had trooped in a body after some more attractive horror, and the inquest obtained no publicity in the halfpenny newspapers. This state of things is especially satisfactory, because the public alarm, which has certainly been excited in some degree by recent fatalities, was not increased, and because the highly technical discussion into which the Court entered ran no risk of being misreported and misrepresented. There was no point of unusual interest in the accident itself, but several emphatic opinions were offered in response to Dr. Waldo's questions upon the relative values of acetone chloroform, of methylated ether and chloroform, and of these drugs when prepared from (dutiable) ethyl alcohol. The anæsthetist had used the latter preparation in this case in accordance with the invariable practice at St. Bartholomew's, where the fatality happened. He told the Court that, in his opinion, ethyl chloroform should always be used in preference to the methylated and acetone products; the latter, owing to their manufacture from non-dutiable spirit, cost about a third of the price charged for the former. In this preference the pathologist concurred. Now this opinion, which prevails strongly at St. Bartholomew's and governs the practice of that hospital, is one from which very many anæsthetists dissent strongly. It is not urged that the best methylated chloroform is superior to the best ethyl chloroform, but simply that the two are, for practical purposes, an identical article and that one is as good as the other. It would be very interesting if a "referendum" of anæsthetists could be taken over the point, not only in London, but throughout the United Kingdom, and such collated wisdom should be based upon clinical experience rather than chemical analysis. In summing up the case for the jury, the Coroner took up the question of the suggested Royal Committee, and of the departmental Home Office inquiry into Coroners' Courts now sitting. He suggested that this important point of the differences between the three kinds of chloroform and the two kinds of ether is one which would profitably engage the attention of either Committee, and the suggestion is one which it requires no expert knowledge of anæsthetics emphatically to endorse. At the same time, there are many more questions equally pressing, and the best endeavours of the leaders of the profession and of the medical Press must be enlisted to obtain a sympathetic reception from the next Government at as early a date as possible. That the issue will be of practical service to the community and the commonwealth we cannot doubt.

